# Survivors of Oncological Disease: Experience and Satisfaction with National Health Care and Service

**DOI:** 10.3390/healthcare13182330

**Published:** 2025-09-17

**Authors:** Tânia Gaspar, Joana Ferreira, Anabela Coelho

**Affiliations:** 1 Psychology, Innovation and Knowledge Service (SPIC), Digital Human-Environment Interaction Labs (HEI-LAB), University Lusofona, 1749-024 Lisbon, Portugal; joana.ferreira040@gmail.com; 2 Comprehensive Health Research Center (CHRC), NOVA University Lisbon, 1600-560 Lisboa, Portugal; anabela.coelho@uevora.pt; 3 Aventura Social—Association, 1649-028 Lisbon, Portugal; 4 Portuguese Laboratory for Healthy Workplaces (LABPATS), 1400-185 Lisbon, Portugal; 5 Escola Superior de Enfermagem São João de Deus, Comprehensive Health Research Centre (CHRC), Universidade de Évora, 7000-645 Évora, Portugal

**Keywords:** survivors of oncological disease, cancer, hospital, experience, satisfaction, psychological follow-up

## Abstract

**Background/Objectives**: The relationship between patients, health professionals, and healthcare organizations is a key factor in patient satisfaction and adherence to care. Organizations, professionals, and patients would benefit from the implementation of organizational measures to promote health literacy and post-discharge psychological counseling. This study aims to explore cancer survivors’ experiences and satisfaction with care, along with identifying their primary needs and barriers. **Methods**: This is a cross-sectional, mixed study with a random and representative sample of the three Portuguese Institutes of Oncology. The patient sample consists of 768 participants, 463 of whom are female (60.3%), aged between 18 and 68 years. **Results**: Most patients reported a positive healthcare experience, particularly regarding staff attention and clarification of doubts, comfort, and ease of access. However, less positive aspects included long waiting times, limited involvement in decision-making, and difficulties understanding medical information. No significant differences were found by gender or age. Overall satisfaction was influenced by the patient’s health status, with those in better health reporting more favorable experiences. **Conclusions**: Patients shared suggestions and complaints about healthcare organization functioning, especially regarding long waiting lists and inadequate conditions during prolonged hospital stays. Overall, their view of the National Health System, particularly Primary Health Care, was less positive compared to the satisfaction with the health organizations under study. This study highlights the importance of follow-up for cancer survivors, with many patients valuing post-discharge contact as a space to share experiences and challenges. The psychological monitoring of patients and families surviving cancer should be clinical practice in health organizations.

## 1. Introduction

Patient satisfaction is widely regarded as a key indicator in assessing the quality of healthcare services, and various reforms have aimed to place the patient at the center of care delivery [1,2]. The increasing age of the patient population poses a significant challenge to healthcare organizations and professionals, as patients are becoming more engaged and seek greater involvement in healthcare decisions and procedures. However, structural aspects such as the type of healthcare funding or service provision account for only a limited portion of overall patient satisfaction [3]. Research indicates that satisfaction with the healthcare system and individual healthcare experiences explains approximately 10% of the variance in patient satisfaction. Other contributing factors include patients’ expectations, physical and mental health status, and personality traits [4,5].

Patients today, as health consumers, expect from the health system what they expect from any other service, i.e., a high-quality service with added value, convenience, adequacy, and respect [6,7]. Sometimes, patients have inappropriate expectations, namely exams, prescriptions, and other medically unnecessary services. Patients tend to be more satisfied if these expectations are met by health professionals. Health professionals, if they adopt an assertive attitude and consultations with enough time to expose and reflect with patients on their concerns, increase patient satisfaction in addition to improving other outcomes [8,9,10].

The quality of the relationship between patients and healthcare professionals is a critical determinant of patient satisfaction, especially in relation to perceived empathy, accessibility, and interpersonal sensitivity [1]. Enhancing this relationship, along with the implementation of organizational strategies to strengthen health literacy, may improve outcomes for both professionals and patients [11].

Patient satisfaction is closely linked to the perceived quality of care, particularly in terms of how well care supports health maintenance or improvement while respecting patients’ needs and values. A person-centered approach to care is fundamental to quality for both ethical and practical reasons. Ethically, it upholds the patient’s right to be treated with dignity and respect when engaging in health services. Practically, person-centered care contributes to more effective service use and improved health outcomes. Within this framework, two key dimensions of quality should be evaluated: patient experience, which encompasses interactions with the healthcare system, and patient satisfaction, which reflects the degree to which care aligns with patient expectations [12].

Patient-centered communication has been shown to enhance the perceived quality of care (QoC), increase patient self-efficacy, and strengthen trust in physicians. Furthermore, greater engagement in one’s care is associated with increased confidence in cancer-related information provided by healthcare professionals [13,14].

Although the overall quality of physician–patient interactions in oncology is generally rated as satisfactory, a significant proportion of patients report a need for more patient-centered communication, as well as more coordinated and comprehensive cancer care [13,14]. Advances in medical care, particularly in early detection and treatment, have significantly contributed to the increase in the number of cancer survivors. Cancer survivorship is commonly described in four distinct phases: the acute survival phase, which begins at diagnosis; the transitional survival phase, marked by the end of treatment and a decrease in contact with the medical team; the extended survival phase, when the survivor remains in remission; and the permanent survivorship phase, during which the individual is cancer-free but may continue to experience long-term physical and psychological effects. This highlights how the impact of cancer often extends well beyond the active treatment period, affecting multiple aspects of a survivor’s life, potentially leading to a decrease in well-being and quality of life [15,16,17,18].

The quality of life of cancer survivors can be categorized into four dimensions: physical well-being and symptoms, psychological well-being, social well-being, and spiritual well-being [19]. Each of these areas will be explored in the following sections.

Many cancer survivors report experiencing symptoms such as anxiety, depression, and cognitive dysfunction. The prevalence of these symptoms may vary depending on the type of cancer or stage of survivorship. For example, a lot of survivors state having memory and/or attention problems, which the literature suggests may be related to previous chemotherapy or certain types of surgery. Persistent worry about the possibility of cancer returning or developing in another part of the body, known as fear of cancer recurrence, can also contribute to ongoing psychological distress. This fear has been linked to reduced quality of life, functional impairments, and increased healthcare costs [19,20,21,22].

In terms of physical well-being and symptoms, cancer survivors commonly report pain and sleep disturbances. Many also express difficulties adjusting to new physical limitations or ongoing health concerns, such as the loss of fertility. Fatigue is a particularly prevalent issue, often associated with prior chemotherapy treatments. Long-term fatigue appears to be especially common among survivors of gastric, bladder, and kidney cancers. Additionally, a decline in physical performance is frequently noted and is linked to factors such as chemotherapy, older age, and gastric cancer. In cases of gastric cancer, this may be intensified by malnutrition and muscle mass loss following gastrectomy [19,22].

Cancer survivorship extends beyond the remission of disease; it encompasses a complex psychological journey marked by emotional, cognitive, and existential challenges. As individuals who have endured life-threatening diagnoses and treatments, survivors frequently experience long-term psychological distress, including anxiety, depression, sleep disturbances, and fear of recurrence. Recent meta-analytic evidence indicates that approximately 24% of survivors continue to experience clinically significant symptoms of anxiety and depression, with sleep disturbances affecting over a third of this population [23]. Qualitative syntheses have further revealed that many survivors struggle with identity disruption, feelings of isolation, and difficulty re-establishing a sense of normalcy in their lives post-treatment [24]. Moreover, the presence of psychological distress is associated with significantly poorer experiences within the healthcare system, including reduced satisfaction with provider communication, lower perceptions of respect, and diminished trust in care quality [25]. These findings underscore the need to prioritize psychological monitoring and support as core components of survivorship care, particularly in healthcare systems striving to deliver patient-centered and holistic oncology services.

Cancer survivors may face significant challenges when returning to work due to the physical changes and psychological distress resulting from diagnosis and treatment, such as fatigue, pain, cognitive deficits, and anxiety. These difficulties can increase the risk of professional exclusion, disability, or early retirement. Returning to work often represents a return to normalcy and can symbolize recovery and resilience, positively influencing mental health and overall quality of life. The survivor’s social well-being can also be influenced by family distress; sexual problems, especially in prostate, ovarian, rectal, bladder and gastric cancer survivors; social isolation; and issues related to body image and appearance, especially in breast cancer survivors [19,22,26,27].

Despite the challenges, some individuals who have overcome cancer may also experience meaningful and positive changes. Post-traumatic growth refers to the constructive personal development that can emerge following a highly demanding life crisis. Research indicates that more than half of cancer survivors report experiencing personal growth because of their cancer journey. Through the resilience built and the coping strategies gained during the active phase of the illness, post-traumatic growth can positively influence overall quality of life [28,29].

These symptoms often improve over time. However, a group of survivors, particularly those who were younger at the time of diagnosis, have a lower socio-economic status, experience persistent fatigue, suffer from lymphedema or arm symptoms, or underwent chemotherapy, are more likely to continue experiencing these symptoms in the long term [30,31,32].

While most cancer research has traditionally focused on patients undergoing active treatment, a growing body of evidence underscores the importance of studying cancer survivors as a distinct population with unique and enduring healthcare needs. Unlike patients in treatment, survivors often continue to experience long-term effects such as persistent fatigue, pain, cognitive impairment, and psychological distress, which significantly impact their quality of life and functional independence [33]. Cognitive dysfunction affects up to 46% of survivors, especially those treated for breast, central nervous system, and hematological cancers, and may persist long after treatment ends. Additionally, survivorship is often marked by heightened fear of recurrence, existential uncertainty, and a challenging process of psychosocial reintegration, including returning to work and resuming social roles. These transitions are frequently described as a struggle to “adjust to a new normal,” involving both physical and emotional adaptation [34]. Survivors also face elevated long-term mortality risks, particularly those treated in childhood, though lifestyle interventions such as maintaining a healthy diet, exercising, and avoiding tobacco have been associated with a reduction in mortality risk. Furthermore, recent studies from European cohorts reveal that survivors make greater use of healthcare services than non-cancer populations, with inequities in access and utilization emerging among those with lower educational levels [35]. Collectively, these findings support the need to address the distinct post-treatment trajectory of cancer survivors, emphasizing long-term monitoring, tailored support services, and survivor-specific research to improve outcomes and healthcare equity.

Even though the literature demonstrates that younger people at diagnosis are more prone to greater distress and poor mental health, older survivors are still a vulnerable population, since some long-time effects of cancer (e.g., hearing trouble, falls, or depression) may be associated with aging and go unnoticed [36].

All this psychological distress can lead to various negative outcomes, including unhealthy behavior, increased use of healthcare services, and reduced treatment adherence, all of which negatively impact the quality of life of the survivors [25].

Even after completing treatment, cancer survivors require ongoing health care, making satisfaction with the health care system crucial. This satisfaction may arise from various aspects of the system. For instance, research indicates that satisfaction with communication from health care providers is linked to fewer comorbidities and a reduced number of office and emergency visits following the treatment period. Additionally, satisfaction with web-based healthcare content plays a significant role, as many survivors turn to the internet for information, self-management, and connecting with other survivors. This online engagement can be an effective strategy for managing and reducing the anxiety and stress commonly experienced in the post-treatment phase [37,38,39].

Survivors’ satisfaction with health care is, therefore, a key factor influencing both health status and quality of life. The literature suggests that patient-centered approaches, such as communication-focused interventions or coping skills training, can enhance satisfaction, strengthen patients’ confidence in managing important aspects of their cancer experience (e.g., disease management), and, eventually, contribute to better quality of life and overall well-being [40,41].

However, there are some factors that may influence satisfaction with the healthcare system of these survivors. Rural cancer survivors seem to have lower satisfaction with the health care system compared to urban survivors, especially related to the accessibility of healthcare professionals and the availability of specialized care when needed [37,42].

The experience the survivors had during the active phase of the illness also influences their satisfaction during the survival phase. Positive experience, characterized by effective communication, sufficient time with providers, and attention to physical and mental health, contributes to satisfaction. On the other hand, the presence of psychological distress and resource constraints can negatively impact this experience. When the patient perceives inadequate attention to their emotional and psychological needs, their satisfaction with healthcare decreases [25].

The literature shows that most of the survivors that referred to low satisfaction with the care they received/are receiving had unmet needs. Abdelsalam and Bayomi [43] reported that the psychological domain had the highest levels of unmet needs among breast cancer survivors. This highlights the need for greater active listening, patient education tailored to individual needs, and appropriate referral systems for social services and psychological support.

The main objective of the study is to understand the experience and satisfaction of cancer survivors, as well as the main needs and barriers identified in the provision of care.

## 2. Materials and Methods

This is a cross-sectional, mixed study with a random and representative sample from the three Portuguese Institutes of Oncology.

### 2.1. Participants

The sample comprised 768 patients, of whom 463 were women (60.3%), with ages ranging from 18 to 68 years. For analytical purposes, participants were categorized into four age groups: 18–24 years (2.3%), 25–44 years (13.3%), 45–64 years (41.5%), and 65 years and older (42.8%). Regarding employment status, 2% identified as students, 38.9% as employed, 8.9% as unemployed, 46% as retired, and 4.3% reported other forms of occupational status.

### 2.2. Instrument

To measure patient satisfaction, the Questionnaire on the Satisfaction of Users of the Health System (QSUSS) [44] was used. Although there are more specific instruments in the field of oncology, since this study is part of a broader study that also assesses satisfaction at other general and university hospitals, we had to opt for an instrument that is recommended by the Directorate-General for Health. The instrument consists of 27 questions; 8 questions are related to sociodemographic data and health care, and 18 of the questions have the following answer options: “yes”; “no”; “not applicable”; and “don’t know/don’t answer”. These questions include “Did you wait more than 4 weeks to have a specialist consultation?”, “Were you satisfied with the time spent by your family doctor/attending physician in the consultation?”, and “Did you feel comfortable and comfortable in contact with the health system?”. The final question, “In your opinion how does the Portuguese Health System work”, is related to the participants’ opinion on the functioning of the Portuguese health system, with the following answer options: “It works well”; “It needs small changes/adjustments”; “It needs major changes/adjustments”; “It needs to be completely restructured”; “does not know/does not answer”. The Global Patient Experience is the total scale that integrates the two dimensions of the scale. 

### 2.3. Procedure

This study was presented to and approved by Ethics Committee of the Lisbon Academic Medicine Centre of the Centro Hospitalar Lisboa Norte of the Faculty of Medicine of the University of Lisbon and obtained a favorable opinion, ref no. 35/19. Following the identification of the target hospitals and the necessary administrative approvals, preliminary meetings were conducted with the clinical directors of the relevant medical specialties to present the research objectives and secure their collaboration in the data collection process. Upon acceptance of the project by hospital administrators and staff, the study was submitted for ethical review to the institutional ethics committees and boards of directors of the three participating hospitals, whose identities will remain confidential. All institutions issued favorable opinions. Once the required approvals were obtained, the data collection phase was initiated.

Regarding the Patient Satisfaction Questionnaire, data collection was carried out by Clinical and Health psychologists through a telephone questionnaire. Each telephone interview had an average duration of 60 min. About 400 patients who used each of the hospitals under study in the last 6 months (information provided by the health organization’s administration) were contacted. The hospitals sent letters explaining the study and informed consent forms to the patients by mail. At the time of telephone contact, the informed consent had been read and only patients who agreed to participate were interviewed. The three hospitals invited to participate in the study, all of which agreed to do so, are oncology hospitals. After obtaining a list of patients who had completed treatment in the last six months, patients were contacted by their respective hospitals, and those who did not refuse to participate were contacted by the team of psychologist researchers. Informed consent was again presented to the patient, and after consent was given, data collection began. This procedure was presented to and approved by the ethics committee and renewed by the ethics committees of each cancer hospital involved.

The cancer hospitals in the study have three levels of care: hospitalization, outpatient care, outpatient consultations, and palliative home care. The patients included in the study at the time of contact receive outpatient follow-up consultations and have already received outpatient care (chemotherapy, for example) and possibly hospitalization (surgical intervention).

No missing data was observed, as all patients who were contacted and consented to participate in the survey responded comprehensively to the questions formulated by the research psychologist. The information was directly entered into a database, and each participant received an identifier number, ensuring anonymity and confidentiality. Open questions were asked about the experience of cancer patients with the care provided by the IPO and the National Health Service as a whole and the barriers identified in patients’ adherence to participation in the study.

Anonymity and confidentiality were strictly maintained across all instruments, as researchers did not have access to any personally identifiable information linked to the collected data. Each participant’s responses were coded using a unique identification number, ensuring the data remained de-identified throughout the process.

Quantitative data obtained through questionnaire responses were analyzed using statistical procedures implemented in IBM SPSS Statistics, version 24.0.

For the analysis of qualitative data, obtained through open questions, content analysis was carried out using the MAXQDA 2020 Program. The qualitative data analysis was performed by analyzing the content of the participants’ discourse. The information was organized into categories and subcategories based on the organization of the discourse.

The interviews were analyzed using qualitative content analysis (QCA) following established methodological guidance. Transcripts were read repeatedly to ensure familiarization, and meaning units relevant to the research questions were identified and coded manually first and then using software. Codes were first generated inductively, remaining close to the participants’ language, and were subsequently organized into broader categories through iterative comparison and abstraction [45]. To enhance the credibility and dependability of the findings, three researchers independently coded all transcripts. After this process, they met to discuss and resolve discrepancies through consensus, thus achieving inter-researcher agreement and reducing potential bias. Negative or deviant cases were also examined to refine categories and strengthen interpretive robustness. Trustworthiness was further ensured by maintaining reflexive notes throughout the process, documenting analytic decisions and assumptions [46].

The results are presented in categories and subcategories, with an analysis of what was said in each of the categories and subcategories conducted by the researchers, followed by illustrative examples with phrases from the participants’ discourse in italics.

## 3. Results

### 3.1. Descriptive Statistics and Internal Consistency of the Dimensions of the Patient Satisfaction Scale

When asked about the classification of their health at that time, 9.5% of the patients classified their health as excellent/good, 71% reported it to be good/fair, and 19.5% reported that their health was poor.

Overall, patients reported having positive healthcare experiences. However, the five most frequently mentioned aspects reflecting less favorable experiences were related to long waiting times, limited involvement in decision-making, and difficulties in understanding the information provided by healthcare professionals. Despite these issues, most patients felt well cared for, experienced comfort during care, had opportunities to clarify their doubts, and did not encounter transportation barriers when accessing consultations (Table 1).

When asked about overall satisfaction, 78.4% of the patients reported satisfaction with the functioning of the health organization under study and 21.6% reported dissatisfaction, with a mean satisfaction value of 1.78 (this value can vary between 1 and 2, with 2 representing lower dissatisfaction with the health organization’s functioning) and a standard deviation of 0.41. Specifically in relation to satisfaction with the functioning of the National Health System, 37.5% reported satisfaction and 62.5% of the patients reported dissatisfaction with the NHS, with a mean satisfaction value of 1.37 and a standard deviation of 0.48.

The items of the Patient Experience Assessment Scale were analyzed through the following steps: (1) Exploratory Factor Analysis with extraction based on the auto value revealed five factors explaining 63.05% of the variance. (2) Exploratory Factor Analysis with the use of Direct Oblimin Rotation revealed two principal factors (Eigenvalue > 2) that explained 40.50% of the variance; factor 1, with an Eigenvalue of 4.63, explained 25.75% of the variance, and factor 2, with an Eigenvalue of 2.66, explained 14.76% of the variance. (3) Internal Consistency Analysis (reliability) for each of the two dimensions obtained acceptable results, with Cronbach’s alpha (α) values of 0.75 and 0.72, respectively, and it did not improve with the exclusion of any of the items.

The items were thus aggregated and organized into two dimensions according to their meaning: one was related to experiences with the care itself and the other was related to access and other financial variables. The patient experience was very positive at a global level and in its dimensions. The internal consistency of the scales was also high (α > 0.71) (Table 2).

### 3.2. Pearson’s Correlations Between Dimensions of the Patient Satisfaction Rating Scale

There is a positive and statistically significant correlation between the Global Patient Experience Scale and the two dimensions. The strongest correlation is between the Global Patient Experience Scale and the Patient Experience in Access/Financial Issues dimension (R = 0.96) and the weakest is between the Global Patient Experience Scale and Patient Experience in General Care (R = 0.35). The two dimensions of the Scale are not statistically correlated (Table 3).

### 3.3. Differences Between Groups—ANOVAs for the Patient Satisfaction Rating Scale

No statistically significant differences were found in patient experience—either overall or across its dimensions—based on gender or age. This includes general care experiences and experiences related to access and financial issues. However, the evaluation of patient experience appears to be influenced by self-perceived health status, with individuals reporting good or fair health tending to report a more positive overall experience (F = 8.76, *p* < 0.001) and a more positive experience in the two dimensions (Patient’s Experience in General Care, F = 6.03, *p* < 0.01; Patient’s Experience in Access/Financial Issues, F = 9.78, *p* < 0.001) than patients with worse health (Table 4).

When the differences were analyzed considering the perception of health and the three health organizations involved, some statistically significant differences were identified.

Patients from Organization C are more satisfied with their global health experience (F = 4.51, *p* < 0.01) and their experience related to general care (F = 5.03, *p* < 0.001) than patients from the other two organizations. Patients from Organization B are the ones who report less satisfaction with their experience when compared to the other organizations (Table 5).

### 3.4. Patient Data Collection Process—Qualitative Results

Regarding the process of collecting data from patients, it is important to highlight some difficulties identified, some positive aspects, and some types of relevant aspects for a better understanding of the results.

#### 3.4.1. Difficulties/Barriers

Some aspects made the process of collecting data from patients difficult. Specifically, there were difficulties in contacting patients because they did not answer the phone, and when they did, they hung up the call.

“*The biggest difficulty I felt was having to call some of the numbers several times until I got them to answer and, even so, there were still people who never answered and who hung up the call.*”

Another difficulty felt was to keep the patient focused on answering the questionnaire; often, the patients expanded on the theme or even addressed other themes unrelated to the questionnaire.

“*Getting the person focused on the purpose of the questionnaire has sometimes proved tricky.*”

“*One of the biggest difficulties in carrying out the questionnaires was the excess of time dedicated to calls with people who were prolonged with subjects unrelated to the topics in question. Sometimes people did not answer what was asked of them and talked about other subjects.*”

Sometimes, difficulties in communicating with patients were felt due to their health conditions and other factors, namely language comprehension or other health problems.

“*Another of the main difficulties felt was the fact that it was necessary to ask questions to individuals who are quite fragile and tired, which in certain situations led to more abrupt (but understandable, of course) responses from them.*”

“*Difficulties in communicating with foreigners or with elderly people who understand almost nothing of what is transmitted.*”

Patients mentioned some suggestions and complaints regarding the survey and the functioning of the health organizations.

As suggestions regarding the survey, they said that it should include open fields for patients to leave their suggestions and that it should include more answer options.

“*There should be a field for observations that users want to leave, since they justify themselves throughout the survey and many ask if they can leave suggestions.*”

“*Many users value more than two answer options (yes or no), since they say that there should be the possibility of an option “sometimes” and that they could justify their answer to be a more complete survey.*”

Regarding the health organization in question, patients referred to difficulties with waiting lists and poor conditions when they spent a lot of time in the hospital.

Some patients had complaints regarding waiting times for scheduling appointments and surgeries, highlighting the fact that the waiting lists are very long and that there is a need for an increase in terms of resources (new equipment) and existing professionals to respond to the long waiting lists.

“*Some patients also showed some dissatisfaction with the conditions of the infrastructures and facilities of the health organization in question, claiming that they are very old.*”

“*It was also possible to verify that some patients revealed the existence of some conflicts with the health organization’s team (namely doctors and nurses), which were, in the meantime, resolved.*”

“*Other patients highlight the fact that it is very costly for them to spend the day in the health organization performing the necessary treatments but recognize that these procedures take time and are actually necessary.*”

“*It was also mentioned by a patient that before, there were volunteers distributing food and drinks during the morning, regretting the fact that this service that brought comfort to users has disappeared.*”

“*Specifically in hospital X, some complaints were mentioned regarding telephone service, the physical condition of the building and parking.*”

In relation to the National Health System in general, including Primary Health Care, the opinion of patients was generally less positive than that in relation to satisfaction with the health organizations under study.

“*With regard to the National Health System, it appears that opinions are not as positive as in relation to the Health Organization in question, and most patients consider that there are several aspects that need to be modified. The waiting time for consultations and the time made available by doctors in health centers are some of the frequent complaints evidenced by patients, and certain users accuse the doctors of health centers of negligence. In addition, some users also mentioned the waiting time in the emergency room as something negative, which needs to be modified.*”

“*In general, patients consider that more technicians (doctors and nurses) are needed in the national health system, that more services should be made available and that there should be a change in the level of facilities in several places.*”

“*Satisfaction with the NHS is generally positive, although users refer to some issues in which the level of satisfaction is lower, namely with regard to the waiting time for scheduling appointments and for service on the day.*”

#### 3.4.2. Positive/Good Practices

Positive aspects were identified in the patient data collection process, which contributed to a better understanding of patient satisfaction and experience.

A large majority of patients had a positive experience with the call, showing a positive, kind, and grateful attitude towards the researchers, and they considered it an opportunity to share their health experience. They highlighted the satisfactory contact with the professionals and collaborators of the health organization and stressed the importance of volunteers.

“*One of the things that surprised me the most was the friendliness of the vast majority of people and how they reacted positively to the call.*”

“*The most significant positive aspect was the availability and friendliness of the users who were interested in participating in and sharing their experiences. There were cases of people who ended up sharing more personal stories and who were very grateful for listening to them. Some of the patients ended up taking advantage of this telephone contact to talk and share a little of their stories and the challenges faced in the face of a disease situation. Another positive aspect is the satisfaction that most reported with the health organizations under study and even the NHS, although many of the people had mentioned some improvements that could be made in the latter.*”

Most patients and family members have a positive experience with the care provided by the health organizations under study.

“*Most people praised the care in the Hospitals, they felt very well treated and cared for by all professionals.*”

“*The patients said that they had been warned by the health organization that they would receive the call and were very willing to participate, which was a positive factor.*”

“*In general, the vast majority of users who agreed to participate in the study said they were very satisfied with the work carried out in the three health organizations under study, considering that they work quite well, and stating that they exceeded their expectations positively. These users stated that they were well cared for by the professionals with whom they contacted, including doctors, nurses and other employees (receptionists, etc.).*”

“*A large number of the patients contacted mentioned the importance of volunteers who carry out important work, stating that they were very available and made the treatment process and contact with the hospital easier.*”

Regarding the gender variable, it was easier to talk to female patients; they were more receptive than male patients, and calls made with male patients were shorter.

In general, in the opinion of patients, family members, and researchers, their perception of the contacts made was very positive, both in terms of adherence to participation in the survey and the level of satisfaction with the National Health System and the health organizations under study. Patients valued the satisfaction survey because it helped them express their experience and because they perceive it as concern on the part of the health organizations under study and the NHS with their opinion and suggestions for improvement.

“*Although, in general, users say they are satisfied with the services provided by the NHS, there are some who consider that there would need to be some changes to further adjust the responses given to existing needs (namely in terms of waiting time for appointments).*”

“*With regard to the service and contact with health professionals, the level of satisfaction is positive. It was notorious to realize that most patients were mostly people belonging to an older age group. And most were also very satisfied with the services provided by the hospital and its employees.*”

“*A situation that happened frequently was that users thanked the contact and felt the need to share a little of their story, not just answering questions directly, but showing a willingness to share a little more of their experiences and experiences regarding the reason why they attend or have attended the hospital and some stories regarding the care received.*”

“*With regard to the general National Health System, it presented more criticism from users compared to the health organizations under study.*”

“*Many of the users are pleased to know that we are carrying out the surveys as there is a “concern” shown with them, validating the action.*”

“*A call was made to a lady, who spent about an hour talking about her life and her problems and who revealed that she was very happy with the fact that she had received the call, because she was able to talk and vent (which highlights the reality that there are individuals who are effectively very isolated and who maintain few social contacts).*”

## 4. Discussion

Overall, patients perceive their healthcare experience as positive. However, five key aspects were identified as contributing to less favorable evaluations, particularly issues related to long waiting times, limited patient involvement in decision-making, and difficulties in understanding the information communicated by healthcare providers. In order to reduce waiting times, some predictive scheduling frameworks and Lean process improvements have been incorporated in specific hospital services, notably improving satisfaction and effectively decreasing waiting times by 22.5 min [47].

Most patients reported feeling well cared for and comfortable with the healthcare services received. They noted that professionals provided opportunities to clarify doubts, and most did not face barriers related to transportation when accessing consultations. No statistically significant differences were observed in the overall patient experience—or in its specific dimensions, such as general care, access, and financial issues—based on gender or age.

However, patient experience appears to be influenced by self-perceived health status: individuals who reported good or fair health tended to describe a more positive overall experience compared to those in poorer health.

Patient satisfaction should be understood as a multidimensional construct. It is shaped not only by the patient–provider relationship and the overall quality of care but also by the broader healthcare system, the patient’s health status, expectations, level of health literacy, and socioeconomic and cultural background [4,9,10].

In line with the other results under study, patient satisfaction in Organization C is more positive, as patients report a better overall health experience and a better experience related to general care than patients in the other two organizations. Patient satisfaction has become increasingly important in the quality of health services provided, as well as in health system reforms [1]. From a systemic and ecological perspective, we see that organizational culture influences the quality of life of professionals and leads to fewer psychosocial risks at work and to a greater involvement of professionals, as well as that more positive and effective processes lead to better economic and financial results, greater satisfaction among professionals, and greater satisfaction among patients [48].

The patient-centered care paradigm increases patient satisfaction and leads to better health outcomes [2,12,49]. Recent evidence also highlights shared decision-making interventions as a key factor in strengthening patients’ trust in clinicians [8] and has been linked to up to a 10% decrease in healthcare expenditures [50]. Patient involvement in decisions further improves satisfaction and adherence, especially among cancer survivors [51].

Our results revealed that “Access/Financial Issues” is uncorrelated with “General Care”. Interestingly, recent evidence suggests that financial or access-related barriers to healthcare do not necessarily correlate with patients’ overall perceptions of healthcare quality. This apparent disconnect may be explained by several interrelated factors. First, the distinction between the structural aspects of access and the interpersonal experience of care plays a crucial role. For example, the OECD’s 2024 PaRIS report found that individuals experiencing financial hardship reported similar levels of person-centered care as those without such hardship across multiple health systems. This indicates that the quality-of-care delivery, particularly in terms of communication, empathy, and respect, can remain high even when access is compromised. Second, the so-called “satisfaction paradox” may also contribute to this phenomenon, as patients often psychologically adapt to constrained circumstances, maintaining a relatively positive evaluation of healthcare services despite facing economic or logistical challenges [52]. Additionally, research on patients with post-COVID-19 conditions in the Netherlands revealed that even when patients encountered access difficulties, they often separated these issues from their evaluations of care quality once they were able to engage with providers. This suggests that perceptions of access and care quality may operate on different cognitive and emotional levels. Taken together, these findings highlight the complexity of healthcare evaluation and suggest that perceptions of care quality are shaped more by the delivery and outcomes of care than by financial or access-related constraints alone [53,54]. This dissociation has important implications for patient satisfaction metrics and policymaking, especially in efforts to improve equity in healthcare delivery.

The literature on the relationship between health status and satisfaction with care is not linear. Most studies establish a negative correlation, with patients with more serious health conditions reporting lower satisfaction with care. However, some studies reveal less clear and linear results [55].

In a study conducted by Hervàs et al. [54], although overall satisfaction levels were high and patients expressed strong loyalty, patients with better health status exhibited higher satisfaction in several subdomains, despite the correlation with overall QoL being statistically unclear. As the study targets cancer survivors who have completed treatment in the last six months, once the imminent risk of death is no longer so present, patients feel greater satisfaction with the professionals and hospital that helped them in their recovery.

The organization that presents the best results is the one that presents the best indicators at the level of the organization, its professionals, and its patients; that is, results in terms of satisfaction and economic and financial aspects have multidimensional contributions, namely the predominant culture of the health organization, the quality of life of the professionals, psychosocial risks at work, especially in terms of interpersonal relationships at work, work–family relationships, and better performance management. All these factors influence the satisfaction of professionals with their work, patient satisfaction, and the economic and financial results of the organization. The results presented allow for a comparison between health organizations and communities as recommended by Andersen [56], Bielecki and Stocki [57], and Braithwaite et al. [47].

The patients had a positive experience in relation to the health organizations under study and a little less of a positive experience in relation to the NHS. However, the indicators that contribute most to dissatisfaction are related to waiting times for consultations and surgeries and involvement in decisions about health care and treatments. Based on national and international recommendations that the patient should be the center of the entire health system [12,58], and from a perspective of meeting the needs and values of patients by providing them with a positive experience and overall satisfaction with the care, access, and health care provided, the patients included in this study reveal the need for improvement in terms of the management of consultations and surgeries, which are aspects that need a systemic approach with a greater focus on the management and administration of hospital organizations. Another aspect that would contribute to better patient satisfaction and experience is the relationship with health professionals in terms of communication, empathy, and greater involvement in decision-making related to health care. In this regard, the organizational culture must be clear about the need for care to be centered on the patient and, consequently, on their needs and their effective involvement in the entire process. On the other hand, this stresses the need to train professionals in terms of communication skills and relationships with the patient [59].

Although an overwhelming 97% of oncology patients report feeling well cared for in surveys, subjective experiences of conflict with healthcare professionals remain common, a paradox that warrants deeper exploration. One factor likely contributing to this discrepancy is the distinction between overall satisfaction, which may reflect institutional or instrumental aspects of care (such as professionalism, timeliness, or technical competence), and specific relational or communication issues that generate tension or conflict at the interpersonal level. For instance, survivors frequently describe breakdowns in communication, such as incomplete information exchange, omission of emotional needs, and lack of care coordination between oncology teams and primary caregivers, that undermine their sense of relational trust, even when they still feel “well taken care of” in broader terms [60].

Furthermore, high emotional and workload burdens on oncology staff, particularly nurses, compromise the depth of patient-centered engagement. While self-assessed patient-centered communication scores remain generally positive, increased burnout is associated with diminished empathy and a reduced capacity to support patient engagement, which may generate frustration or perceived conflict, even when patients continue to feel technically cared for. Cultural and systemic factors also shape this tension. Patients may hesitate to openly criticize or challenge professionals due to perceived power imbalances, social expectations, or institutional norms [61].

In addition to its important contributions, this study has its limitations. There are limitations in terms of the quality and accuracy of responses provided over the telephone. The absence of body language and of a controlled environment makes it difficult to interpret and explore certain responses in depth, reducing the richness of qualitative data. There may be a desire to please the interviewer or an omission of sensitive information, especially on delicate topics such as pain, prognosis, quality of life, or mental health. Studies show that telephone interviews, compared to face-to-face interviews, tend to generate more socially desirable responses.

As this paper is part of a broader study involving the proposal of a mathematical model for managing healthcare organizations with multiple informants, patient satisfaction was converted into a binary form to enable analysis. However, the use of a complementary qualitative methodology helps to better understand the values resulting from quantitative methodologies, even if they are binary.

For reasons of confidentiality, anonymity, and personal data protection, the specific information about cancer-related variables (type, stage, treatment) were not available to the research team, and they could be useful to contextualize the results.

In healthcare settings, attitudes held by both professionals and patients diverge markedly between emergency or life-threatening scenarios (such as advanced-stage cancer) and routine consultations. In emergency contexts, clinical staff often experience time pressure and emotional strain, which can degrade communication quality and reduce the frequency of serious illness discussions, as shown in oncology, where documentation of such conversations significantly decreases as the clinic day progresses due to decision fatigue and scheduling constraints [62].

Moreover, when treating older cancer patients, healthcare professionals’ own attitudes, shaped by experience, specialty background, and systemic limitations, play a substantial role in guiding decision-making, sometimes in lieu of strong empirical evidence, with potential consequences for alignment with patient values. By contrast, during routine primary care appointments, discrepancies often arise between what patients expect and what professionals perceive. These contrasts underscore a critical divide: emergency and serious illness settings demand empathetic, patient-centered communication under duress, whereas routine care hinges on aligning preventive medicine strategies with patient expectations and clarifying misalignments between provider assumptions and patient desires [63].

A conclusion of the present study is that a follow-up of cancer survivors should be carried out; most of the patients contacted reported that contact was very important and that they could share their experience and barriers. The psychological monitoring of patients and families surviving cancer should be clinical practice in health organizations.

These conclusions are in alignment with recent European multicenter research. The ABC study observed over 95% follow-up coverage and noted that, while satisfaction was moderate (50%), survivors identified crucial areas for improvement, namely psychological support, enhanced provision, better management of late effects, and assistance with work-related issues [64]. Systematic reviews of survivors’ preferences underscore the value of continuity of care, provider familiarity, and individualized communication modes in enhancing satisfaction [65]. In North America, survivors receiving survivorship care plans demonstrated significantly greater confidence in their primary care providers and reported improved satisfaction and trust [66]. Although UK survivors generally had comparable long-term quality of life and healthcare utilization to matched controls, disparities in certain psychosocial outcomes persisted, emphasizing the potential of structured support to address lingering survivor needs [67]. Together, these findings support the integration of follow-up protocols that are comprehensive, personalized, and psychosocially oriented. Such systems not only support survivor well-being and quality of life but also foster trust, satisfaction, and engagement in post-treatment care, essential components of high-quality, patient-centered oncology services in Portugal.

## 5. Conclusions

The conclusion of the present study is that a follow-up of cancer survivors should be carried out. Most of the patients contacted reported that contact was very important and that they could share their experience and barriers. The psychological monitoring of patients and families surviving cancer should be clinical practice in health organizations.

Final recommendations:

1. Waiting Times: Patient satisfaction is more influenced by subjective perceptions (expected vs. actual wait) rather than just objective wait [68]. Managing expectations via real-time updates helps reduce dissatisfaction. AI-driven and dynamic “standby” scheduling systems in the NHS have begun shortening surgical waitlists and reducing cancelations. To enhance the overall quality of oncology care, it is recommended that health systems prioritize reducing waiting times for treatment initiation and optimize the physical and logistical conditions of outpatient cancer services. Patients frequently report prolonged waiting periods in poorly equipped environments, often without access to basic necessities such as adequate seating, privacy, and nutritional support during long infusion sessions. These factors contribute to patient distress and may negatively impact treatment adherence and satisfaction. Ensuring dignified, supportive, and patient-centered outpatient settings, particularly in chemotherapy day units, is essential for improving the care experience and promoting equity in oncology services [69].
2. Communication, Empathy, and Patient Involvement: Enhanced training in communication and shared decision-making (SDM), such as decision aids, improves understanding, autonomy, and patient satisfaction [70].
3. Organizational Culture: A clear organizational focus on patient-centered care, empathetic interactions, and ethical deployment of SDM is crucial for embedding patient-focused care [48].
4. Psychological Support in Oncology: Given the high prevalence of psychological distress among cancer patients and survivors, it is strongly recommended that psychosocial support be systematically integrated into oncological care pathways. Psychological screening and counseling services should be offered not only during active treatment but also throughout survivorship, where issues such as fear of recurrence, fatigue, and identity disruption are particularly pronounced. Furthermore, targeted support should be extended to family members, who often face emotional and caregiving burdens with limited professional guidance. The implementation of structured, multidisciplinary survivorship programs, including mental health professionals, is essential to improving long-term outcomes, patient well-being, and the overall quality of cancer care [71].
5. Professional Development: Continuous training in interpersonal communication and shared decision-making is essential [59].
6. Support for Rural Cancer Survivors and Those with Low Health Literacy: To promote equitable survivorship outcomes, tailored psychosocial and educational support must be prioritized for cancer survivors residing in rural areas and those with limited health literacy. These populations face heightened challenges, including reduced awareness of survivorship care plans, difficulties accessing exercise and nutrition guidance, and transportation barriers, all of which impede recovery and quality of life. Interventions should include locally accessible programs or e-health solutions for nutritional counseling, structured physical activity, and peer or professional support groups, combined with clear, plain-language educational materials to help survivors understand and adopt healthy lifestyle changes. Engaging local primary care providers and community networks to deliver these services can enhance accessibility and effectiveness in underserved settings [72].

## Figures and Tables

**Table 1:** Descriptive statistics of the items of the Patient Satisfaction Scale.

Patient Experience	No %	Yes %
1. Have you waited more than 4 weeks to have a specialist consultation?	63	37
2. Did you wait more than 1 h on the day of the appointment to be seen by the doctor?	54.4	45.6
3. Did you miss appointments because you didn’t have transportation?	97.5	2.5
4. Missed appointments due to financial difficulties?	96.2	3.8
5. Have you stopped undergoing medical examinations, treatments or follow-up appointments due to financial difficulties?	94.5	5.5
6. Have you stopped purchasing prescribed medication due to financial difficulties?	94.1	5.9
7. In general, were you satisfied with the time spent by the doctors in the consultation?	5.8	94.2
8. Were you satisfied with the time spent by your family doctor/attending physician in the consultation?	6.9	93.1
9. In general, did the doctors give you the opportunity to clarify your doubts?	5.5	94.5
10. Did your family doctor/attending physician give you the opportunity to clarify your doubts?	6.2	93.8
11. In general, did you notice everything the doctors told you?	8.0	92.0
12. Did you understand everything your family doctor/attending physician told you?	6.4	93.6
13. In general, have doctors involved you in decisions about health care and treatment?	9.6	90.4
14. Has your GP/treating physician involved you in decisions about healthcare and treatment?	13.5	86.5
15. Did the quality of the services provided correspond to expectations?	6.2	93.8
16. Did you feel comfortable and comfortable in contact with the health system?	3.0	97.0
17. Did you feel a lack of privacy during the medical appointment?	90.9	9.1
18. Did you feel well taken care of by the professionals you contacted?	2.2	97.8

**Table 2:** Descriptive statistics and internal consistency of the dimensions of the Patient Satisfaction Scale.

Patient Satisfaction	M	DP	α
Global patient experience	1.90	0.11	0.71
Patient experience in general care	1.89	0.13	0.75
Patient experience in access/financial issues	1.96	0.15	0.72

**Table 3:** Pearson’s correlations between the dimensions of the Patient Satisfaction Rating Scale.

Patient Experience	Global Patient Experience	
Global patient experience		
Patient experience in general care	0.35 **	
Patient experience in access/financial issues	0.96 **	0.06

Note. ** *p* < 0.001.

**Table 4:** ANOVA—differences considering the perception of health between the dimensions of the Patient Experience Assessment Scale and Satisfaction Assessment Scale.

Patient Experience	Good/Fair Health		Poor Health		F
	M	SD	M	SD	
Global patient experience	1.91	0.10	1.87	0.13	8.76 ***
Patient experience in general care	1.89	0.12	1.86	0.16	6.03 **
Patient experience in access/financial issues	1.97	0.13	1.92	0.22	9.78 ***

Note. *** *p* < 0.001, ** *p* < 0.01.

**Table 5:** ANOVA—differences between health organizations in the dimensions of the Patient Experience Assessment Scale and Satisfaction Assessment.

Patient Experience	Organization A		Organization B		Organization C		F
	M	SD	M	SD	M	SD	
Global patient experience	1.90	0.09	1.88	0.11	1.91	0.11	4.51 **
Patient experience in general care	1.88	0.10	1.86	0.14	1.90	0.13	5.03 ***
Patient experience in access/financial issues	1.95	0.13	1.95	0.17	1.96	0.14	0.60 (n.s.)

Note. *** *p* < 0.001, ** *p* < 0.01.

## Data Availability

The original contributions presented in this study are included in the article. Further inquiries can be directed to the corresponding author.
